# Advancements in Understanding Spasticity: A Neuromusculoskeletal Modeling Perspective

**DOI:** 10.3390/jcm14228092

**Published:** 2025-11-15

**Authors:** Mohammad S. Shourijeh, Argyrios Stampas, Shuo-Hsiu Chang, Radha Korupolu, Gerard E. Francisco

**Affiliations:** Department of Physical Medicine and Rehabilitation, UTHealth Houston McGovern Medical School, Houston, TX 77030, USA

**Keywords:** spasticity, neuromusculoskeletal modeling, reflex hyperexcitability, muscle-tendon mechanics, computational modeling, gait analysis, cerebral palsy, stroke rehabilitation, spinal cord injury

## Abstract

Spasticity, a complex consequence of upper motor neuron lesions, poses challenges for clinical assessment due to its neural and mechanical origins. Traditional scales like the Modified Ashworth and Tardieu Scales provide subjective, context-limited insights, often missing spasticity’s dynamic nature. Neuromusculoskeletal (NMS) modeling offers objective, quantitative insights by integrating patient-specific muscle–tendon properties, reflex dynamics, and multi-joint biomechanics. This scoping review examines advancements in spasticity modeling, comparing mechanical, neurological, and integrated approaches, and their applications in conditions like cerebral palsy and stroke. We highlight barriers to clinical translation, including computational demands and regulatory challenges, and propose future directions, such as real-time simulation and machine learning integration, to enhance personalized assessment and treatment.

## 1. Introduction

Spasticity is a complex sensorimotor disorder characterized by involuntary muscle hyperactivity in the presence of central paresis. While historically defined as a velocity-dependent increase in muscle tone due to hyperexcitable stretch reflexes, more recent frameworks broaden this concept to include rigidity, dystonia, spasms, and sensory abnormalities, reflecting the multifaceted nature of motor dysfunction following upper motor neuron injury [1,2,3]. This involuntary overactivity results not only from altered spinal reflex pathways but also from impaired voluntary motor control, maladaptive plasticity, and sensory disinhibition due to cortical and subcortical damage [1].

Spasticity is prevalent across a spectrum of neurological conditions, affecting approximately 25–40% of stroke survivors, 84–86% of individuals with multiple sclerosis (MS), 48–87% of those with spinal cord injury (SCI), and 69–80% of people with cerebral palsy (CP) [4,5]. A significant proportion develop severe or disabling spasticity, up to 79% in SCI, 47% in MS, and 9–10% post-stroke, resulting in complications such as pain, contractures, joint deformities, pressure ulcers, impaired mobility, falls, and sleep disturbances. These issues contribute to increased healthcare utilization, long-term disability, and reduced quality of life [4,5,6]. Functionally, spasticity impairs both posture and movement, often limiting independence. In CP, for instance, it can cause crouch gait and restrict ambulation [7], while in stroke survivors, it frequently affects upper limb function, interfering with activities of daily living such as dressing, grooming, and feeding [8,9]. Despite its clinical significance and widespread impact, the pathophysiology of spasticity remains only partially understood, posing ongoing challenges for the development of effective and individualized interventions. Emerging definitions that encompass the full clinical spectrum and recognize neuroplastic and sensory components provide a more comprehensive foundation for therapeutic targeting and patient-centered Management [1].

**Figure 1:**
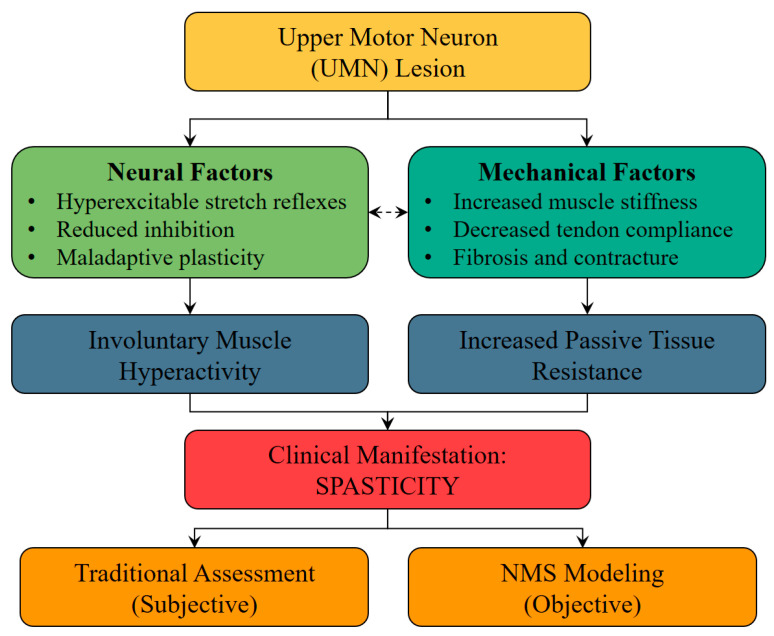
Conceptual pathways linking UMN lesions to spasticity. An upper motor neuron lesion gives rise to two interacting pathways: neural factors (hyperexcitable, velocity-dependent stretch reflexes with reduced inhibition and maladaptive plasticity) and mechanical factors (increased muscle stiffness, decreased tendon compliance, and fibrosis and contracture). These pathways produce involuntary muscle hyperactivity and increased passive tissue resistance, which together manifest clinically as spasticity. Traditional scales (e.g., MAS, TS) reflect a subjective composite of both pathways, whereas neuromusculoskeletal (NMS) modeling provides an objective decomposition of neural versus mechanical contributions.

The complexity of spasticity arises from its dual neural and mechanical origins (Figure 1). Neural factors include hyperexcitability of stretch reflex pathways, reduced inhibitory control, and altered proprioceptive feedback, while mechanical factors encompass increased intrinsic muscle stiffness, altered tendon compliance, changes in the extracellular matrix, and joint contractures. These factors interact dynamically, varying across individuals, conditions, and even specific tasks, making standardized assessment and treatment difficult.

Traditional clinical tools, such as the Modified Ashworth Score (MAS; Table 1) and Tardieu Scale (TS; Table 2), rely on subjective evaluations of muscle tone and reflex responses. They often fail to distinguish neural from biomechanical contributions (i.e., passive tissue resistance vs. reflex-mediated active muscle force) and struggle to separate spasticity from related impairments such as rigidity or fixed contractures [10,11,12,13]. This subjectivity can compromise treatment planning, for example, through suboptimal botulinum toxin dosing, imprecise injection site selection, or generic orthotic design, which reduces treatment effectiveness, increases the risk of functional decline, and perpetuates reliance on assessment tools that limit diagnostic and therapeutic precision. Incorporating objective biomechanical modeling approaches may help address these challenges by providing more detailed insights into the underlying neural and mechanical impairments; when used alongside clinical judgment, such models can enhance assessment precision, support more informed intervention strategies, and help anticipate patient-specific treatment responses (e.g., functional improvements following interventions) [6].

Early modeling efforts included mechanical models focusing on passive tissue properties [12,14], neurological models simulating reflex pathways [15,16], and threshold control models quantifying reflex triggers such as the Tonic Stretch Reflex Threshold (TSRT) and Dynamic Stretch Reflex Threshold (DSRT) [17,18,19]. While these approaches provided valuable insights, they often operated in isolation, lacking the integration needed to capture spasticity’s complexity during functional tasks such as walking or reaching. For instance, mechanical models could not replicate neurally driven phenomena such as clonus, while neurological models often ignored biomechanical constraints (e.g., realistic muscle force-generating capacities, moment arms, or segmental inertial properties), reducing their clinical applicability.

The advent of NMS modeling has marked a significant leap forward, enabling integrated simulations of neural control and musculoskeletal dynamics [20,21]. These models generate physiologically realistic representations of movement, reflex behavior, and resistance to stretch, offering a comprehensive framework to study spasticity. By incorporating patient-specific data, such as muscle geometry, reflex thresholds, and biomechanical properties (e.g., muscle optimal fiber lengths, tendon slack lengths, pennation angles, and segment inertial parameters), NMS models support personalized simulations that enhance clinical relevance. For example, a personalized model for a child with CP might simulate how increased gastrocnemius stiffness and heightened reflex sensitivity contribute to crouch gait, guiding targeted interventions such as botulinum toxin injections or selective dorsal rhizotomy [22,23,24].

Computational platforms such as OpenSim (open-source; [25,26]) and the AnyBody Modeling System (commercial; [27]) have laid the groundwork for advanced biomechanical analyses and simulations by enabling the estimation of joint kinematics, muscle forces, and movement dynamics across a variety of tasks. Building on these foundations, open-source frameworks like Moco [28] and SCONE [29] have introduced predictive simulation and optimal control capabilities, allowing researchers to simulate how movement might change in response to altered neuromuscular or musculoskeletal conditions. Collectively, these tools can be applied to spasticity and have enhanced the ability to model impaired motor control and optimize interventions virtually. Workflows such as the Neuromusculoskeletal Modeling (NMSM) Pipeline [30,31] and SimCP [32] integrate physics-based modeling with patient-specific parameter estimation and predictive simulations and can replicate stretch-induced responses with high fidelity. NMSM comprises two main modules: model personalization and treatment optimization.

The model personalization module estimates patient-specific parameters for joints, muscle–tendon units, and neural control rules via muscle synergy theory, while the treatment optimization module optimizes treatment goal parameters, such as reduced spasticity at a joint or improved bilateral arm function, by iteratively adjusting treatment parameters, including botulinum toxin dosage or robotic rehabilitation adaptive level of assistance. Similarly, the Calibrated EMG-Informed Neuromusculoskeletal Modeling System (CEINMS; [33]) leverages EMG-driven modeling to personalize muscle–tendon parameters in a manner comparable to NMSM’s capabilities. Its real-time extension, CEINMS-RT [34], enables fast, real-time assessments of neuromuscular dynamics, making it suitable for time-sensitive clinical applications. These platforms support the extraction of spasticity-specific metrics such as joint stiffness and damping, as well as reflex thresholds such as TSRT and DSRT. These quantitative biomarkers exceed the diagnostic granularity of traditional clinical scales, enabling more precise assessment and individualized treatment planning. For instance, impedance-based metrics can quantify dynamic joint resistance during gait, informing orthotic design and guiding targeted interventions.

Despite these advancements, clinical adoption remains limited by several barriers: the computational complexity of high-fidelity models, the need for extensive validation across diverse patient populations and clinical contexts, the lack of user-friendly interfaces for clinicians, and regulatory hurdles related to medical device approval and risk classification. These challenges hinder the transition from research to practice. Emerging technologies, including machine learning, wearable sensors, and multiscale modeling, offer promising solutions to enhance model scalability, usability, and clinical integration.

This review synthesizes the current state of spasticity modeling, identifies key methodological advancements, and proposes future directions, with a focus on personalized NMS approaches. We aim to differentiate these models from traditional frameworks, highlight their capacity for patient-specific simulation, and explore their potential to advance clinical assessment and treatment. Key enabling technologies, such as hybrid modeling, real-time sensor integration, and artificial intelligence (AI)-driven personalization, are discussed, alongside future directions like regulatory validation and translational applications in rehabilitation robotics. By contextualizing these advancements, we underscore the transformative potential of personalized NMS modeling in improving diagnostic accuracy, therapeutic precision, and patient outcomes across diverse neurological conditions.

## 2. Overview of Spasticity Modeling Approaches

### Mechanical, Neurological, and Threshold Control Modeling

Computational modeling of spasticity has historically followed three primary paths: mechanical, neurological, and threshold control models [22]. Each approach targets distinct aspects of spasticity, passive biomechanical properties, reflex-mediated neural responses, and reflex thresholds, respectively, offering valuable insights but facing limitations when applied in isolation, particularly for simulating complex spastic behavior across diverse individuals and functional tasks.

Mechanical models represent muscle–tendon units and joints using simplified elements such as springs and dampers to capture stiffness, viscosity, and resistance. Foundational studies by Fee and Foulds (2004) [14] and Alibiglou et al. (2008) [12] employed viscoelastic analogs in pendulum tests to quantify increased muscle tone and altered damping in spastic limbs. More recent advancements by Ang et al. (2018) [35] and Le Cavorzin et al. (2001) [36] incorporated nonlinear tissue behaviors to reflect responses under varied movement velocities and joint angles, enhancing accuracy for passive assessments [22]. Some models have even attempted to characterize the distinct phases of a spastic catch (e.g., pre-catch, catch, and post-catch) to better represent the dynamic evolution of joint resistance. These models excel in simulating static or low-velocity conditions, such as joint resistance during clinical examinations, but fail to capture neural-driven phenomena like velocity-dependent reflex contractions or clonus. For example, mechanical models cannot replicate the dynamic interplay of reflex hyperexcitability and muscle activation observed during gait, limiting their applicability to functional tasks.

Neurological models focus on the central and peripheral mechanisms underlying reflex responses, modeling stretch reflexes with threshold-based activation and neural gain parameters. Koo and Mak (2006) [15], de Vlugt et al. (2010) [16], and Shin et al. (2020) [37] advanced these models by incorporating muscle spindle feedback and reflex loop delays, improving simulations of velocity-dependent behaviors. These models have elucidated how altered reflex thresholds and delayed inhibitory responses contribute to spastic movement dysfunction, such as exaggerated elbow flexion in stroke survivors. However, their reliance on idealized or population-averaged parameters often overlooks individual variability. Moreover, by neglecting biomechanical context, such as joint mechanics, muscle–tendon compliance, or segmental inertia, neurological models produce abstract simulations that lack biomechanical fidelity for tasks such as reaching or stair climbing, limiting their ability to predict how neural changes manifest as altered joint torques or movement patterns.

Threshold control models, developed by Levin and Feldman (1994) [17], Calota et al. (2008) [18], and Bar-On et al. (2013) [19], emphasize reflex thresholds like TSRT and DSRT, which quantify the joint angle or velocity at which reflex activity is triggered. Applied in stroke and CP [38,39,40], these models provide insights into neural hyperexcitability and have been used to assess spasticity during passive and active movements. For instance, TSRT measurements can identify the elbow angle at which a reflex is elicited, helping clinicians determine which muscles exhibit heightened reflex sensitivity and should be targeted for botulinum toxin injections to maximize therapeutic effect [18,39,40]. Conceptually, these thresholds can define a patient-specific ‘spastic joint space’, illustrating the range of motion compromised by hyperreflexia. However, these models require integration with biomechanical data to simulate functional tasks accurately. Without accounting for muscle–tendon dynamics, joint constraints, and the inertial properties of the limb segments, they may not fully capture spastic behavior during complex movements such as gait.

Recognizing these limitations, early hybrid models sought to combine mechanical and neural elements. Kamper et al. (2001) [13] integrated stretch reflex thresholds with intrinsic muscle stiffness to simulate reflex-mediated torques during limb motion, demonstrating how increased reflex gain amplifies joint resistance. These models highlighted the value of integration yet remained low-dimensional and nonpersonalized, limiting them to simple test scenarios (e.g., single-joint pendulum tests) and thereby laying the conceptual groundwork for more comprehensive approaches.

Table 3 compares the main modeling approaches, summarizing their features, strengths, limitations, and clinical applicability. The shift toward personalized NMS modeling directly addresses the key shortcomings of isolated methods, namely, limited integration of neural and mechanical components and insufficient personalization. By unifying these domains, NMS models enable realistic simulations of spastic behavior under functional movement conditions, supporting more accurate diagnosis and precision rehabilitation across diverse patient populations.

## 3. Neuromusculoskeletal Modeling in Spasticity

NMS modeling provides a structured framework to simulate the interactions among neural control, musculoskeletal structure, and biomechanical response in pathological states characterized by spasticity [22,24,41]. Unlike isolated mechanical or neurological models, NMS approaches integrate anatomical fidelity, muscle dynamics, and reflex behavior within a unified computational environment, which can help address spasticity’s multifactorial nature. These models represent the interplay of neural hyperexcitability, altered muscle–tendon properties, and joint mechanics, offering a systematic approach to studying spasticity across conditions. The recent shift toward personalized NMS simulations, which incorporate patient-specific parameters, has strengthened their potential to estimate individual motor responses and to generate hypotheses that may inform clinical decision-making in research and early translational contexts [24,30,33,41]. For example, simulations have been used to suggest which muscles may contribute most to gait abnormalities in cerebral palsy, with the possibility of informing choices such as targeted botulinum toxin injections or orthotic prescriptions [19,24]. Such applications support more individualized and potentially more effective treatment planning.

### 3.1. Dynamic Neuromuscular Models

Early NMS models established the foundation for dynamic neuromuscular simulations by examining reflex responses and intrinsic muscle properties during limb motion. He et al. (1997) [42] developed one of the first frameworks to incorporate reflex thresholds and gain parameters into pendulum test simulations, reproducing the abnormal oscillations characteristic of spastic limbs. This work demonstrated the feasibility of integrating biomechanical and neural feedback within a closed-loop framework, paving the way for later, more detailed modeling approaches.

Subsequent studies, such as those by Fee and Foulds (2004) [14], introduced velocity-dependent feedback mechanisms to simulate muscle responses to rapid stretch, a hallmark feature of spasticity. These developments enabled reproduction of clinically observable behaviors, such as the sudden “catch” described in TS. Later work by Koo and Mak (2006) [15] and Falisse et al. (2018) [23] expanded this architecture to include proprioceptive feedback from muscle spindles and Golgi tendon organs, allowing simulations of both excitatory and inhibitory influences on muscle activation. These refinements improved the physiological realism of simulated responses under varying movement speeds and joint configurations.

Recent dynamic neuromuscular models increasingly emphasize personalization by incorporating subject-specific muscle geometry, activation patterns, and reflex characteristics [30,43]. For example, a personalized model for a stroke survivor may estimate how heightened reflex sensitivity in the biceps influences elbow flexion during reaching, potentially informing targeted interventions such as botulinum toxin injections. Embedding reflex pathways within the broader context of muscle–tendon mechanics and segmental inertia enhances anatomical and physiological fidelity [23,44]. This integration is important, since reflex behavior depends not only on neural thresholds but also on the instantaneous length and velocity of the muscle–tendon unit, which are shaped by joint kinematics, external forces, and the muscle’s own force–length–velocity properties. Recent evidence further suggests that muscle spindle firing may be closely correlated with muscle force generation (e.g., [44]).

The clinical relevance of dynamic neuromuscular models lies primarily in their ability to quantitatively link neural and mechanical mechanisms underlying spasticity [45,46]. These models can assist in research-oriented diagnostics by estimating reflex hyperexcitability, support treatment planning through predictive simulations, and generate hypotheses about intervention outcomes [30,45,47]. For instance, simulations may explore how reducing reflex gain through pharmacological intervention could influence gait symmetry in cerebral palsy. As personalization methods continue to advance, such models are expected to contribute increasingly to individualized assessment and rehabilitation planning across diverse patient populations.

### 3.2. Physics-Based Simulations

Recent advances in physics-based neuromusculoskeletal (NMS) analyses and simulations, implemented in platforms such as OpenSim [25,26], the AnyBody Modeling System [27], OpenSim Moco [28], SCONE [29], SimCP [32], CEINMS-RT [34], and NMSM Pipeline [30], have markedly enhanced the realism and analytical power of spasticity modeling. These frameworks use explicit biomechanical formulations, including automated generation of equations of motion, detailed muscle–tendon modeling, and complex joint representations, to capture dynamic interactions among neural control, musculotendon mechanics, and limb movement. Using forward dynamics with reflex-augmented neuromuscular controllers, physics-based simulations can reproduce velocity-dependent spastic responses across joint configurations, movement speeds, and loading conditions. Parameter optimization further personalizes these models by identifying subject-specific reflex gains, thresholds, and muscle–tendon properties that characterize the severity and distribution of spasticity. In turn, optimal control frameworks allow predictive exploration of how modifying these parameters, such as reducing reflex gain or altering muscle stiffness, might influence spastic behavior during functional tasks, supporting treatment planning and intervention design. This integration of physics-driven computation with subject-specific data enables exploration of how altered muscle–tendon properties or reflex dynamics affect movement, advancing the capacity of NMS modeling to study spasticity under physiologically realistic conditions.

Falisse et al. (2020) [41] used personalized physics-based models to simulate crouch gait in children with CP, showing that altered muscle–tendon properties, such as reduced compliance or excessive passive stiffness, often contribute more to functional impairment than spasticity alone. These findings help distinguish neural from mechanical contributions to abnormal movement, informing decisions about interventions such as tendon lengthening or orthotic support. Similarly, van der Krogt et al. (2016) [24] employed subject-specific neuromusculoskeletal simulations of instrumented hamstring stretch tests in children with CP, allowing separation of neural components (spasticity) from mechanical factors (contracture-related stiffness) in measured torque responses.

A relevant feature of physics-based NMS modeling is the ability to estimate joint impedance, defined by stiffness as resistance to displacement [48,49,50,51] and damping as resistance to velocity. These variables provide objective, quantitative measures of spasticity-related mechanical behavior and complement subjective clinical scales such as MAS. In model-based analyses, joint stiffness and damping are computed from partial derivatives that isolate how joint torque changes with joint angle and joint angular velocity while holding other state variables, such as muscle activation and muscle–tendon history, fixed. These are often called true stiffness and true damping because they reflect intrinsic mechanical properties under controlled activation conditions. By contrast, quasi-stiffness and quasi-damping are slopes obtained from measured joint angle–torque and angle–torque–rate relationships during movement. They combine the effects of passive tissues, active muscle forces, and ongoing changes in activation [50]. Detailed mathematical formulations for these impedance measures are provided in Supplementary Material S1. Depending on the task and measurement protocol, quasi measures may also include reflex-mediated torque, which is one reason they tend to overestimate resistance compared with true measures. Clinically, these measures are valuable because they enable estimation of dynamic changes in joint stiffness and damping across tasks and activation states, capturing the variable presentation of spasticity. For example, simulations can quantify increased knee stiffness in a CP patient during stair climbing, providing insights for orthotic design and adjustment, an objective measure not discernible from MAS scores alone.

Complementary to these mechanical metrics, neural measures such as the Tonic Stretch Reflex Threshold (TSRT) and Dynamic Stretch Reflex Threshold (DSRT) quantify the joint angles and velocities at which stretch-induced muscle activation occurs [17,18,19]. TSRT reflects reflex activation during slow stretches, while DSRT characterizes responses during rapid movement, both offering critical information about the neural components of spasticity. Integrating these neural metrics with biomechanically derived impedance parameters allows NMS models to represent both intrinsic mechanical changes (e.g., increased passive stiffness, altered tendon properties) and neurophysiological contributors (e.g., reflex thresholds), providing a more comprehensive understanding of spasticity mechanisms.

### 3.3. Clinical Applications

The translation of NMS modeling into clinical practice is an emerging area characterized by growing interest and early-stage implementation. Personalized computational frameworks are increasingly supporting assessment, intervention planning, and treatment monitoring, particularly in research settings and pilot clinical applications (e.g., [36,44]). These frameworks have the potential to provide objective, quantitative measures of spasticity-related impairments, surpassing the limitations of subjective scales such as MAS and offering clinicians actionable insights.

A critical clinical challenge is differentiating between contracture (a primarily mechanical limitation due to fixed muscle and/or tendon shortening or joint stiffness) and spasticity (neural-driven hyperreflexia with mechanical consequences). Traditional scales such as MAS and TS often conflate these phenomena, as both present as increased resistance to passive stretch. However, their treatment pathways differ substantially: contractures typically require surgical intervention (e.g., tendon lengthening) or prolonged stretching protocols, while spasticity responds to pharmacological agents such as botulinum toxin or antispastic medications [52,53].

Van der Krogt et al. (2016) [24] demonstrated how NMS modeling addressed this misclassification in children with CP undergoing hamstring assessments. By simulating instrumented stretch tests, their models separated neural contributions (reflected in velocity-dependent reflex activity and TSRT values) from mechanical factors (captured by passive tissue stiffness). In cases where MAS scores suggested severe spasticity, modeling revealed that contracture-related stiffness dominated the torque response, leading clinicians to prioritize progressive lengthening exercises or surgical tendon lengthening over botulinum toxin injections. This distinction prevented ineffective pharmacological treatment and accelerated functional improvement. Similarly, Falisse et al. (2020) [41] used physics-based simulations to show that altered muscle–tendon properties, rather than reflex hyperexcitability alone, primarily drove crouch gait in specific CP patients, redirecting treatment from spasticity management toward correcting musculoskeletal deficits through orthotic support or surgery.

Botulinum toxin remains a cornerstone of neural spasticity management, but its effectiveness depends critically on precise muscle targeting and appropriate dosing. Suboptimal injection site selection or dosing errors can result in incomplete spasticity reduction, functional decline due to excessive weakening of adjacent muscles, or transient benefits requiring frequent re-injection [54,55,56]. Ang et al. (2018) [35] developed an upper-limb NMS model combining motion capture data with surface electromyography (sEMG) to estimate muscle activation patterns in stroke survivors. By comparing simulated results with clinical spasticity scores, they demonstrated that NMS modeling could identify abnormal co-contraction during reaching, such as simultaneous hyperactivity of the biceps and triceps, that traditional scales missed. This precision enabled targeted botulinum toxin delivery to the biceps while sparing the triceps, improving elbow extension range of motion without compromising flexor strength needed for activities of daily living. In contrast, MAS-guided injections might have treated both muscles indiscriminately, reducing functional capacity.

Similarly, threshold control models using TSRT and DSRT have guided injection site selection by quantifying which muscles exhibit heightened reflex sensitivity at specific joint angles [18,38]. For instance, TSRT measurements in ankle plantar-flexors can identify whether the gastrocnemius or soleus dominates spastic resistance during gait. A model might reveal that the gastrocnemius reaches reflex threshold at 5° dorsiflexion while the soleus remains quiescent until 15°, suggesting preferential gastrocnemius targeting to preserve soleus function for push-off power. Such granularity exceeds the capabilities of ordinal scales, which assign a single score to the entire muscle group.

Orthotic prescriptions for spasticity management, e.g., ankle–foot orthoses, traditionally rely on clinician experience and trial-and-error adjustments. NMS models can simulate how different orthotic stiffness levels or ankle joint configurations affect gait mechanics, supporting evidence-based design. A model might predict that a rigid ankle–foot orthosis reduces knee flexion moment in a CP patient with crouch gait, improving upright posture but simultaneously increasing hip extensor demand, risking compensatory lordosis [57]. This insight allows clinicians to balance orthotic support with muscle capacity, optimizing functional outcomes.

In surgical planning, NMS simulations have been used to predict post-operative gait changes following procedures such as hamstring lengthening or rectus femoris transfer [32]. Falisse et al. (2020) [41] demonstrated that personalized models could predict whether a child with CP would benefit from tendon lengthening (if passive stiffness dominated) versus selective dorsal rhizotomy (if reflex hyperexcitability dominated). This predictive capacity potentially reduces surgical failure rates and avoids unnecessary interventions, directly improving patient outcomes compared with decisions based solely on clinical examination.

Advanced laboratory and wearable measurement technologies enhance the objectivity and precision of NMS models beyond what subjective scales can achieve. Portable kinematic sensors such as inertial measurement units (IMUs) and electrogoniometers capture joint motion outside traditional gait laboratories, while dynamometers quantify torque resistance during both passive and active movements [39]. EMG has long been used to characterize reflex thresholds such as TSRT and DSRT, which quantify the joint angle and velocity at which reflex activity is triggered [17,18,58,59]. This approach provides an objective means of assessing neural hyperexcitability by identifying the onset of reflex activation in specific muscles during controlled stretch tests. More recently, wearable EMG systems have begun to enable similar assessments in clinical and ambulatory environments [60,61], potentially supporting more accessible and patient-specific evaluations that can inform botulinum toxin dosing or surgical decision-making.

Advanced imaging techniques, such as magnetic resonance imaging (MRI) and ultrasound elastography, provide complementary insights into muscle architecture and mechanical properties. MRI characterizes muscle cross-sectional area and tissue composition, while ultrasound elastography quantifies in vivo muscle stiffness and fascicle behavior, facilitating calibration of model parameters. Barber et al. (2011) [62] combined ultrasound and dynamometer assessments to show that young adults with spastic cerebral palsy exhibit significantly increased ankle joint stiffness, reduced gastrocnemius fascicle strain, and smaller muscle cross-sectional area compared with typically developing individuals, findings that informed model-based predictions of passive resistance contributions. Similarly, Lacourpaille et al. (2014) [63] demonstrated the potential of ultrasound shear-wave elastography to quantify muscle stiffness, establishing its clinical applicability for neuromuscular disorders and providing a methodological foundation for its later use in spasticity modeling.

NMS simulations also play a critical role in predicting intervention outcomes. Personalized models have been used to evaluate the effects of orthopedic surgeries (e.g., tendon lengthening or transfers) [41], pharmacological treatments (e.g., botulinum toxin injections) [64], and rehabilitative strategies (e.g., robotic-assisted therapy) [65,66]. Quantitative metrics derived from such models, including estimated changes in muscle viscosity or reflex thresholds (DSRT), have been used to objectively track treatment efficacy, sometimes revealing improvements not captured by subjective clinical scales [35,38]. These simulations help clinicians identify interventions likely to yield functional gains while avoiding those that may exacerbate impairments due to individual biomechanical constraints, such as excessive passive stiffness. In addition to evaluating outcomes, emerging frameworks increasingly use optimization algorithms to adjust treatment parameters, such as injection dosage, orthotic stiffness, or robotic assistance level, to achieve specific therapeutic goals, extending the role of NMS models from assessment toward active treatment planning.

Spasticity manifests differently across neurological conditions, and neuromusculoskeletal simulation frameworks have the potential to capture these condition-specific profiles [24,67]. In stroke, increased stiffness is often accompanied by reduced damping, leading to joint instability and exaggerated oscillations during movement [52,53,68]. In contrast, cerebral palsy (CP) may involve abnormal co-contraction patterns and excessive reflex gain, resulting in stiff, uncoordinated movements [69,70]. By accurately representing these distinct profiles, such models can support the design of targeted therapies that address the underlying causes of motor dysfunction [25,41]. For instance, a model developed for a patient with CP might suggest selective dorsal rhizotomy to reduce reflex hyperexcitability [71], whereas a stroke-specific model might prioritize botulinum toxin treatment to mitigate reflex-mediated activity and intrinsic stiffness [54].

Wearable technologies and real-time feedback systems further extend the clinical potential of NMS modeling. Closed-loop therapy environments can integrate models with sensor data (e.g., EMG, kinematics, or force measurements) to track spastic muscle behavior and provide clinicians or robotic devices with actionable feedback. For example, a wearable system might update model parameters in real time during a gait training session, adjusting robotic assistance to optimize muscle activation patterns using frameworks such as CEINMS-RT [34]. Although still largely in the research phase, this degree of personalization holds promise for accelerating rehabilitation, reducing clinician workload, and enhancing patient engagement through immediate, task-specific feedback [72].

Emerging joint impedance estimates, when obtained through personalized simulations, represent a high-potential class of quantitative biomarkers. These metrics, including dynamic stiffness and damping, complement qualitative scales such as MAS by providing continuous, task-relevant measures of spasticity that capture both neural and mechanical contributions. Unlike MAS, which assesses resistance during passive movement, impedance-based metrics can quantify stiffness and damping during functional activities, offering insight into spastic behavior under realistic task conditions. For example, impedance estimates can characterize how spasticity affects elbow flexion during a reaching task, guiding the design of assistive devices or therapy protocols. By embedding these estimates within NMS frameworks, clinicians gain access to high-resolution, interpretable metrics that support real-time decision-making and long-term treatment planning, ultimately improving patient outcomes.

## 4. Comparing and Evaluating Models

As spasticity models grow in complexity and diversity, a rigorous framework for comparison and evaluation is essential to assess their utility and guide future development. Models range from simple mechanical representations to sophisticated, personalized NMS frameworks, each judged by their fidelity to real-world phenomena, predictive accuracy, clinical usability, and scalability across populations. This section reviews the primary criteria for evaluating spasticity models, explores how NMS models address these benchmarks, and discusses the implications of current limitations for patient care. A summary comparison table (Table 3) is provided to highlight key characteristics, strengths, and limitations of each modeling approach.

### 4.1. Metrics for Evaluation

The primary goal of spasticity models is to accurately replicate clinical and experimental observations, encompassing passive stretch responses, joint stiffness profiles, and complex dynamic behaviors such as gait deviations, co-contractions, and clonus. A key benchmark for evaluating model fidelity is the degree of agreement between simulated outputs and experimental measurements that capture neuromuscular and biomechanical behavior, such as EMG activity (reflecting neural activation), joint torques (reflecting mechanical response), and kinematic trajectories (reflecting movement patterns). Falisse et al. (2018, 2020) [23,41] demonstrated strong correlations between simulated and observed EMG patterns in CP patients during passive limb movement and gait, highlighting the importance of neural feedback mechanisms in achieving realistic activation timing. Similarly, experimental measurements of joint kinetics and kinematics, obtained through tools such as dynamometers, motion capture systems, or wearable sensors, have been used to evaluate model accuracy, ensuring that simulated outputs reproduce the characteristic features of spastic responses observed during functional tasks such as walking or reaching [41,44].

Given the substantial inter-individual variability in spasticity, parameter sensitivity and robustness are critical for clinical applicability. Factors such as lesion location, lesion severity, age, resting muscle tone, and habitual activity level influence spastic behavior, requiring models to accommodate variation in reflex gain, passive muscle stiffness, tendon slack length, muscle fiber length, and reflex activation thresholds. Sensitivity analyses (e.g., Kamper et al., 2001 [13]) show that small perturbations in input parameters can materially alter model outputs, underscoring the need for accurate, individualized calibration. For example, a pediatric cerebral palsy model should incorporate developmental changes in muscle architecture, whereas a post-stroke model should reflect age-related declines in neural control.

Scalability and generalizability pose additional challenges, particularly for early models focusing on single joints or limbs. Recent NMS models have progressed to simulate full-body movements across diverse tasks, such as sit-to-stand transitions or stair climbing [26]. However, generalizability depends on access to diverse anatomical datasets and well-characterized biomechanical parameters. Population-specific differences in muscle architecture (e.g., fiber lengths, pennation angles, physiological cross-sectional areas), spasticity patterns, and neural compensation strategies must be considered to ensure clinical utility across pediatric and adult cohorts and different neurological diagnoses. Personalized NMS models, built around patient-specific data from motion capture, EMG, and MRI, offer a promising solution, providing high-fidelity representations of individual spastic behavior and intervention responses.

### 4.2. Integration of Neural and Biomechanical Components

The most effective spasticity models integrate neural dynamics and biomechanical properties into a cohesive framework, reflecting the physiological reality of spastic movement. On the neural side, features like TSRT, DSRT, feedback delays, and excitatory/inhibitory balance are essential for simulating velocity-dependent behaviors. For instance, Falisse et al. (2018) [23] showed that incorporating proprioceptive feedback improves the reproduction of clinically observed EMG activation patterns, particularly for exaggerated stretch reflexes and clonus in spastic muscles. These neural elements capture the dynamic interplay of reflex pathways and motor control, which is critical for understanding spasticity’s neural origins.

An accurate representation of muscle–tendon stiffness (both active and passive components), joint damping, and passive resistance from non-contractile tissues is also essential biomechanically [24,73]. Advances in personalized modeling enable subject-specific estimation of these properties, moving beyond generic approximations to clinically meaningful biomarkers. Impedance modeling, which defines joint stiffness and damping as context-sensitive, time-varying quantities, has emerged as a powerful method for quantifying spastic behavior during dynamic tasks [13,50,61]. Unlike traditional quasi-stiffness metrics, NMS-based formulations isolate mechanical contributions from neural activation, providing interpretable measures of intrinsic muscle and joint properties.

Reflex threshold metrics such as TSRT and DSRT complement biomechanical measures by providing a neural perspective on spasticity. Derived from EMG and kinematic data during passive movements, these thresholds quantify the onset of reflex activity and offer insights into altered neural control strategies [17,18,19]. When integrated into NMS models, they enhance model fidelity, enabling more accurate predictions of how spasticity interacts with joint position, velocity, and mechanical loading. For example, a model incorporating TSRT can simulate how reflex hyperexcitability influences ankle dorsiflexion in cerebral palsy, guiding surgical or pharmacological intervention strategies.

The integration of neural and biomechanical components within personalized NMS models represents a major advancement, enabling high-fidelity simulations that capture the full spectrum of physiological processes underlying spasticity. Such models can support a multidimensional understanding of spastic behavior, facilitating precise diagnosis and individualized treatment planning across diverse clinical populations.

## 5. Gaps and Future Directions

Despite advancements, several critical gaps in spasticity modeling remain and must be addressed to fully realize its clinical and research potential. Personalized NMS modeling has made progress in simulating spasticity and supporting early-stage clinical assessments. However, its broader adoption as a routine clinical tool will require further improvements in clinical usability, validation diversity, regulatory pathways, and computational efficiency before expanding into more visionary applications. These limitations collectively hinder the adoption of objective, simulation-informed tools in clinical practice. Subjective assessments such as the MAS fail to distinguish between neural and biomechanical contributors, leading to suboptimal treatment choices, for example, inappropriate surgical timing or poorly designed orthoses, which ultimately reduce therapeutic effectiveness and patient quality of life. This section identifies the most pressing limitations, examines their implications for diagnosis and treatment, and proposes a staged roadmap that prioritizes actionable near-term solutions while outlining longer-term transformative opportunities.

### 5.1. Bridging Research and Clinical Practice: Immediate Priorities

The usability gap between research-grade NMS models and clinical practice limits their impact on patient care and represents a significant barrier to widespread adoption. These models have the potential to revolutionize spasticity management by improving diagnostic accuracy, predicting intervention outcomes, and tracking therapeutic progress. For instance, NMS models can differentiate neural and biomechanical contributions to spasticity, enabling precise diagnoses that prevent the misclassification of contractures (a primarily mechanical limitation) as spasticity (a neural-driven phenomenon with mechanical consequences). They also have the potential to predict the outcomes of interventions like tendon lengthening or botulinum toxin injections, ensuring treatments are tailored to individual needs. Additionally, quantitative metrics from models can objectively monitor changes in spasticity severity, guiding therapy adjustments and enhancing long-term outcomes.

However, current shortcomings, reliance on subjective scales like MAS, computational complexity, and limited clinical usability, hinder these benefits. Without objective tools, clinicians may struggle to distinguish reflex-driven hyperactivity from passive stiffness, risking inappropriate treatments such as mistargeted botulinum toxin injections or ineffective orthotic prescriptions. For example, two patients may receive similar MAS scores despite having different underlying impairments, reflex hyperexcitability in one and increased passive stiffness in the other, leading to mismatched interventions, such as suboptimal botulinum toxin targeting that addresses the wrong impairment mechanism. These errors can exacerbate motor impairments, delay recovery, and diminish quality of life.

Computational complexity is a major barrier to real-time or near-real-time applications. High-fidelity NMS simulations, incorporating multiscale muscle properties (e.g., Hill-type formulations with detailed force–length–velocity characteristics or even fiber-level representations), neural dynamics, and finite element methods, are computationally demanding, requiring significant processing power and time. This computational demand limits their use in fast-paced clinical environments, where timely decision-making is critical. In the absence of accessible real-time tools, clinicians must often rely on coarse, subjective measures like MAS, which may not accurately differentiate between neural and mechanical contributors to spasticity.

Similarly, complex models requiring technical expertise limit clinicians’ ability to utilize objective tools, perpetuating reliance on less precise methods. While NMS modeling platforms are becoming more sophisticated, they often require technical expertise in programming, data processing, and numerical optimization, making them inaccessible to most clinicians. This gap prevents the adoption of objective, quantitative measures that could improve the accuracy and consistency of clinical assessments and treatment decisions. Specific usability barriers include the complexity of OpenSim’s scripting interface for nonprogrammers, which requires Python or MATLAB proficiency, and the calibration workflows in NMSM Pipeline, SimCP, and CEINMS, which demand advanced parameter tuning and biomechanical expertise. These challenges have hindered adoption even in research hospitals with computational resources.

To enhance the clinical utility of NMS models, it is critical to balance their technical complexity with accessibility for clinicians who may lack expertise in computational modeling or biomechanics. The current technical focus of NMS models, while essential for researchers, risks alienating clinicians, limiting adoption in routine practice. Incorporating clinical examples and case studies can illustrate how models translate to actionable insights, making their value tangible.

Consider a personalized NMS model for a 10-year-old child with cerebral palsy presenting with crouch gait. Traditional MAS assessment might assign a grade 2 score for ankle plantar flexion, indicating a “more marked increase in muscle tone through most of the range of motion,” yet this measure does not provide muscle-level specificity; for example, it cannot distinguish which plantar flexor muscles contribute most to the resistance. Moreover, this ordinal measure cannot determine whether the observed resistance stems primarily from heightened reflex sensitivity (spasticity), increased passive muscle stiffness (contracture), or both. A personalized NMS simulation integrating the child’s motion capture data, EMG, and ultrasound-derived muscle architecture reveals that the gastrocnemius exhibits a DSRT of 5° plantar flexion during rapid stretch (indicating early reflex triggering) combined with elevated passive fiber stiffness from shortened optimal fiber length. By simulating botulinum toxin injection scenarios, the model predicts that targeting the gastrocnemius medial head with 3 units/kg would reduce reflex-mediated torque by 40% during the stance phase of gait, resulting in a predicted 15° increase in knee extension at initial contact and improved gait symmetry, outcomes not discernible from the MAS score alone. Furthermore, the simulation suggests that combining this pharmacological intervention with a progressive stretching protocol addressing passive stiffness would yield greater functional gains than botulinum toxin alone. This case example demonstrates how NMS models guide precise, multimodal interventions, enhancing clinician confidence in their application.

Similarly, in a stroke survivor with elbow flexor spasticity affecting reach-to-grasp tasks, an NMS model might reveal abnormal co-contraction of biceps and brachioradialis during voluntary extension attempts, a pattern obscured by MAS testing, which only assesses passive resistance. By quantifying each muscle’s contribution to flexor torque throughout the reaching movement, the model identifies the biceps as the dominant contributor at mid-range angles (60–90° flexion) while the brachioradialis dominates at end-range. This granular insight enables targeted botulinum toxin delivery to the biceps while sparing the brachioradialis, preserving flexor strength needed for activities of daily living while improving extension range of motion. Such case-based translation makes the clinical value of NMS modeling concrete and actionable.

Addressing computational complexity and usability requires practical solutions, such as developing user-friendly platforms or prototypes tailored to clinical workflows. Short-term solvable approaches include surrogate modeling and model-order reduction techniques, which approximate key model outputs more efficiently while preserving predictive accuracy. Machine learning-based surrogate models, trained on high-fidelity simulation data, can deliver near-instantaneous predictions suitable for clinical workflows. Existing tools, like simplified interfaces in OpenSim or prototype dashboards for the CEINMS-RT framework, offer promising starting points. These platforms could integrate automated data processing (e.g., EMG, motion capture) and provide intuitive visualizations, such as predicted joint torque profiles or reflex threshold maps, to support real-time decision-making.

For example, a clinician-facing dashboard might display simulated outcomes of botulinum toxin injections, highlighting target muscles (e.g., gastrocnemius medial head vs. lateral head) and expected functional gains (e.g., “15° increase in knee extension during stance phase”). The interface could present comparative scenarios side by side, such as injection alone versus injection combined with progressive stretching, allowing clinicians to select evidence-based multimodal strategies. Color-coded muscle activation maps during gait could visualize which muscles exhibit excessive reflex-mediated activity versus passive resistance, making complex biomechanical data interpretable at a glance.

Building on this concept, future extensions of NMS modeling could support more integrated rehabilitation planning by incorporating therapy parameters alongside pharmacological interventions. Since the effectiveness of botulinum toxin depends heavily on the frequency, timing, and type of rehabilitation that follows [55,56,74,75,76], such models could provide decision support for post-injection therapy. For instance, a model might recommend initiating intensive task-specific training around 4–6 weeks post-injection, when spasticity reduction is near its peak [77], or suggest optimal repetition frequencies for stretching exercises tailored to the patient’s passive stiffness profile.

Short-term solvable issues include developing intuitive interfaces, automated data integration pipelines, and seamless sensor integration, technically feasible with current software engineering capabilities. User interface design is equally critical. Current modeling tools require expertise in coding and biomechanics, which limits their use in routine clinical care. Developing intuitive, clinician-facing applications with simplified data entry, automated integration of patient-specific data (e.g., EMG, motion capture, imaging), interactive visualization dashboards, and decision-support modules will lower adoption barriers. By prioritizing intuitive design and automated workflows, these tools can reduce the technical barrier, enabling clinicians to leverage NMS models for precise diagnosis, intervention planning, and treatment monitoring.

Long-term efforts should focus on sustainable models for maintaining user-friendly clinical platforms, clinician training in basic modeling principles, and integration with electronic health record systems. However, long-term systemic challenges remain, particularly the need for extensive validation of reduced-order models across diverse patient populations and clinical scenarios to ensure they maintain fidelity to the full models they replace. Interdisciplinary collaboration among engineers, clinicians, neuroscientists, and data scientists is essential to define use cases, establish performance benchmarks, and co-develop tools that meet clinical needs to ensure usability and sustainability in real-world workflows.

### 5.2. Validation and Standardization: Building Clinical Trust

Validation and standardization are critical for clinical adoption, ensuring models are robust, reliable, and trusted by clinicians and regulators. Limited sample diversity in validation studies restricts model generalizability and raises the risk of inaccurate characterizations and poorly tailored interventions for underrepresented groups, such as pediatric patients or those with SCI. Many simulations are based on small, homogeneous datasets from single clinical centers or specific subpopulations, limiting applicability across broader demographics. This lack of diversity can result in models that fail to account for variations in spasticity presentation or underlying biomechanical differences due to age, sex, or pathology, potentially leading to inaccurate characterization of impairments or suboptimal treatment choices for underrepresented groups, such as pediatric patients or those with atypical etiologies like SCI.

Large-scale validation studies, testing NMS models across diverse populations with varying etiologies, functional levels, and demographics, are essential. Expanding validation datasets through multicenter collaborations and establishing open-access repositories that aggregate diverse patient data represent practical, near-term steps. Open-access datasets and multicenter collaborations will facilitate the sample sizes and heterogeneity needed for robust validation. Long-term progress will depend on sustained funding, institutional support, and formalized data-sharing frameworks that enable robust validation across the full spectrum of spasticity presentations.

“Is my model good enough?” Standardized benchmarks must be established to evaluate model performance, including agreement with clinical scales (e.g., MAS, TS), task-specific functional outcomes (e.g., changes in gait speed, joint range of motion, or work performed at a joint), responsiveness to intervention, and computational efficiency. The lack of standardized benchmarks hinders model comparison and clinical trust. Standardized movement tasks, such as instrumented gait, reaching, or sit-to-stand trials, can enable cross-study comparisons and inform best practices. For example, a benchmark might require models to predict changes in gait symmetry post-intervention with at least 80% agreement with observed outcomes, ensuring clinical relevance. Consensus on such benchmarks would allow different modeling approaches to be compared objectively, accelerating identification of best practices and building clinician confidence in model reliability.

Short-term priorities include establishing consensus benchmarks for model performance (e.g., agreement with MAS/TS, sensitivity to therapeutic change, and task-specific functional outcomes) and ensuring transparency in reporting model assumptions and limitations. For instance, developers should clearly document the populations on which models were trained and validated, the range of spasticity severities for which predictions are reliable, and known failure modes (e.g., inaccurate predictions for patients with severe contractures or atypical movement patterns).

Regulatory compliance is equally crucial as computational models progress toward classification as medical devices or decision-support systems. For computational models to be accepted as medical decision-support tools, they must undergo rigorous validation against gold-standard clinical measures and comply with regulatory guidelines from bodies such as the Food and Drug Administration (FDA) and the European Medicines Agency (EMA). Adherence to FDA and EMA guidelines mandates transparent reporting of model assumptions, parameter ranges, validation procedures, and limitations.

Specific regulatory challenges include uncertainty about how AI-driven or machine learning-enhanced models will be classified, as medical devices requiring full premarket approval, or as clinical decision-support tools subject to less stringent oversight. Regulatory uncertainty also affects AI-driven and machine-learning-enhanced models, which may be variably classified as medical devices requiring full premarket approval or as decision-support tools with lighter oversight. The FDA’s evolving guidance on software as a medical device (SaMD) creates ambiguity that discourages developers from pursuing regulatory pathways.

In the longer term, coordinated efforts among developers, clinicians, regulatory bodies, and professional societies will be needed to create standardized evaluation frameworks and streamline approval processes for validated models. The lack of regulatory validation impedes the integration of objective tools, obstructing accurate diagnosis and targeted treatment. Coordinated efforts are essential to expedite the pathway from simulation to clinical impact, ensuring models promote equitable care across diverse populations.

### 5.3. Personalized Modeling: Leveraging Advanced Data Sources

Once foundational usability and validation milestones are achieved, personalized NMS modeling can be further enhanced through integration of advanced imaging and sensor data. Personalized NMS modeling represents a transformative approach that tailors simulations to individual neuromechanical characteristics, thereby improving assessment accuracy and intervention outcomes. A key priority is integrating advanced imaging data, such as MRI, diffusion tensor imaging (DTI), and ultrasound elastography, to inform anatomical and physiological parameters. These techniques provide high-resolution insights into muscle architecture (e.g., volumes, optimal fiber lengths, pennation angles), tissue composition (e.g., fat infiltration, fibrosis), passive mechanical properties (stiffness from elastography), and neural tract integrity, enabling precise calibration of variables such as muscle–tendon length, cross-sectional area, and stiffness. For example, DTI can map neural connectivity in stroke patients, refining models of reflex hyperexcitability, while ultrasound elastography quantifies muscle stiffness in CP, validating impedance parameters and informing passive force components of muscle models.

Real-time integration of wearable sensor data is another critical area. Wearable technologies that capture sEMG, joint kinematics, and ground reaction forces enable dynamic model updates that support adaptive rehabilitation strategies. For instance, a wearable system might track sEMG signals during gait training, updating a model to optimize robotic assistance in real time. Machine learning algorithms can enhance this process by identifying trends in sensor data, predicting episodes of increased spasticity, or suggesting therapy adjustments. These capabilities are essential for continuous monitoring and personalized intervention. However, gaps in sensor integration and data processing risk incomplete personalization, leading to suboptimal outcomes, such as ineffective orthotic adjustments or pharmacological dosing.

Reflex threshold-based modeling, such as TSRT and DSRT, further enriches personalization by quantifying patient-specific reflex dynamics. These thresholds, derived from EMG and kinematic data, define the onset of stretch reflexes on a per-muscle basis, enabling simulations that reflect individual neural profiles. Embedding these thresholds within NMS models enhances fidelity, supporting multidimensional spasticity assessments. Investigating these thresholds during both passive and active movements can reveal crucial differences, as volitional effort can modulate reflex sensitivity and the functional range of motion. For example, a model incorporating DSRT might simulate how reflex hyperexcitability affects wrist extension in a stroke patient, guiding targeted botulinum toxin injections. Furthermore, model refinements could also explicitly account for the influence of the initial stretch level or joint position at the onset of movement, as this has been shown to modulate spastic reflex responses but is not always incorporated into current models. Addressing gaps in imaging, sensor integration, and comprehensive reflex characterization is crucial to ensure accurate diagnosis and tailored treatments, maximizing therapeutic effectiveness.

### 5.4. Emerging Technologies: Long-Term Vision

Once the foundational challenges of usability, validation, and personalization are addressed, emerging technologies can expand the scope and impact of spasticity modeling. These longer-term opportunities represent transformative directions that build upon near-term successes.

Machine learning offers powerful tools for personalization and automation in neuromuscular modeling. Supervised models can estimate parameters from sparse datasets, while reinforcement learning can optimize intervention strategies in silico before clinical application. For example, a predictive model trained on longitudinal patient data might anticipate spasticity changes, enabling proactive therapy adjustments to prevent functional decline. Such models can also approximate complex musculoskeletal dynamics, allowing rapid prediction of impedance profiles or reflex thresholds from wearable sensor data and bypassing computationally intensive full-model simulations during routine monitoring.

Multiscale modeling represents another frontier, bridging microscopic processes (e.g., sarcomere dynamics, cross-bridge cycling, neural firing patterns) and macroscopic outcomes (e.g., joint movement, gait) [78,79]. These models capture interactions across biological scales, providing insights into how cellular abnormalities propagate to affect motor function. For instance, a multiscale model might simulate how altered neural firing in CP contributes to muscle co-contraction, informing targeted pharmacological therapies. While computationally complex, multiscale approaches enhance the biological realism of NMS models, supporting precision medicine. However, such models should be pursued after establishing robust validation frameworks for current NMS approaches, ensuring that added complexity translates to clinically meaningful improvements.

Augmented Reality (AR) and Virtual Reality (VR) technologies complement NMS modeling by delivering immersive, context-specific motor tasks. Combined with personalized models, AR/VR environments can simulate patient-specific movement challenges, offering therapists real-time insights into motor deficits and adaptive strategies. For example, a VR system might simulate a CP patient’s gait, allowing therapists to test orthotic adjustments virtually before physical implementation. These technologies are particularly valuable in pediatric and post-stroke rehabilitation, where engagement and task realism are critical to therapy success. AR/VR integration represents a promising long-term direction once core modeling platforms achieve clinical maturity and widespread adoption.

## 6. Conclusions

This scoping review synthesized current developments in neuromusculoskeletal modeling of spasticity, emphasizing the integration of neural and biomechanical components and the challenges of clinical translation. Neuromusculoskeletal modeling provides a powerful framework for quantifying the complex neural and biomechanical interactions underlying spasticity. By integrating patient-specific data from motion capture, EMG, and imaging, these models enable individualized simulations that can reveal the relative contributions of reflex hyperexcitability, passive stiffness, and altered muscle mechanics. Such mechanistic insights can inform surgical planning, optimize botulinum toxin targeting, and guide rehabilitation strategies beyond the limits of traditional qualitative assessments.

Despite substantial progress, translating NMS models into routine clinical practice will require streamlined computational pipelines, standardized validation protocols, and regulatory pathways that ensure reliability and transparency. Achieving this goal depends on sustained collaboration among engineers, clinicians, and regulatory bodies, along with the establishment of large, diverse datasets for model calibration and testing.

Ultimately, the integration of validated, user-friendly NMS platforms into clinical workflows could transform spasticity management from a primarily descriptive discipline into a predictive and quantitative science, enabling precision rehabilitation tailored to each individual’s neuromechanical profile.

## Figures and Tables

**Table 1:** Modified Ashworth Scale.

Grade	Description
0	No increase in muscle tone
1	Slight increase in muscle tone, minimal resistance at end of range of motion (ROM)
1+	Slight increase in muscle tone, catch followed by minimal resistance through less than half of ROM
2	More marked increase in muscle tone through most of ROM, but affected part easily moved
3	Considerable increase in muscle tone, passive movement difficult
4	Affected part rigid in flexion or extension

**Table 2:** Tardieu Scale: The Tardieu Scale assesses spasticity by evaluating muscle responses to passive movements at varying velocities. V1 (slow) measures passive range of motion without triggering the stretch reflex, V2 (medium, limb falling under gravity) detects mild spastic responses, and V3 (fast) elicits velocity-dependent spasticity. Muscle reaction is graded 0–4, from no resistance (0) to sustained clonus (4), with a “catch” indicating a sudden increase in tone at a specific angle. The angle of catch, measured in degrees via a goniometer during V2 or V3, quantifies the spastic threshold. Fatigable clonus (Grade 3, <10 s) or non-fatigable clonus (Grade 4, >10 s) indicates severe spasticity.

Velocity of Movement	Quality of Muscle Reaction (Grade)	Description	Angle of Catch (R1)/PROM (R2)
V1: Slow (as slow as possible)	N/A (or “No spastic reaction expected”)	Baseline measurement of passive range of motion (PROM) under minimal stretch reflex activation.	R2 (Angle of full PROM) is recorded. No R1 (catch) is expected.
V2: Medium (limb falling under gravity)	A grade (0–4) is assigned based on the observed muscle response.	Assesses muscle response to stretch at a moderate speed. A catch (R1) indicates spasticity.	R1 (Angle of catch) is recorded if present.
V3: Fast (as fast as possible)	A grade (0–4) is assigned based on the observed muscle response.	Assesses muscle response to stretch at a fast speed. Elicits velocity-dependent spasticity (catch/clonus).	R1 (Angle of catch or clonus) is recorded if present.

**Table 3:** Summary Comparison of Spasticity Modeling Approaches.

Model Type	Key Features	Strengths	Limitations	Clinical Applicability	Example Applications
Mechanical	Spring–damper analogs, passive tissue modeling	Simple to implement; effective for capturing passive stiffness	Does not model neural dynamics; limited to low-velocity tasks	Passive assessments, e.g., pendulum tests	Pendulum tests for elbow stiffness
Neurological	Reflex pathways, neural gain, feedback delays	Simulates neural contributions; useful for studying reflexes	Lacks biomechanical realism; often population-averaged parameters	Understanding reflex hyperexcitability	Identifying reflex triggers in stroke
Threshold Control	TSRT/DSRT reflex thresholds based on joint angle/velocity	Quantifies reflex triggers; applicable during passive movements	Requires biomechanical integration for task-level simulation	Botulinum toxin targeting; spasticity quantification	Optimizing injection sites in CP
Hybrid	Combines neural and mechanical elements	Simulates reflex–mechanical interactions	Often low-dimensional; not fully personalized	Simulated resistance during clinical tasks	Modeling elbow catch in stroke
Personalized NMS	Patient-specific anatomy, EMG, multiscale modeling	High anatomical fidelity; predicts functional outcomes	Computationally intensive; requires technical expertise	Diagnosis, treatment planning, outcome prediction	Gait optimization in CP

## Supplementary Materials

The following supporting information can be downloaded at: https://www.mdpi.com/article/10.3390/jcm14228092/s1.

## Abbreviations

The following abbreviations are used in this manuscript:

NMS	Neuromusculoskeletal
MAS	Modified Ashworth Scale
TSRT	Tonic Stretch Reflex Threshold
DSRT	Dynamic Stretch Reflex Threshold
EMG	Electromyography
IMU	Inertial Measurement Unit
MRI	Magnetic Resonance Imaging
DTI	Diffusion Tensor Imaging
AI	Artificial Intelligence
AR	Augmented Reality
VR	Virtual Reality
CP	Cerebral Palsy
SCI	Spinal Cord Injury
MS	Multiple Sclerosis
