# Supplementary material for: Advancements in Understanding Spasticity: A Neuromusculoskeletal Modeling Perspective
Source: J Clin Med. 2025 Nov 15;14(22):8092. doi: 10.3390/jcm14228092 (PMC12653318; doi:10.3390/jcm14228092)
Supplement: Supplementary file 1 [file jcm-14-08092-s001.zip › jcm-3855195-supplementary.pdf]

Advancements in Understanding Spasticity: A Neuromusculoskeletal Modeling Perspective

Mohammad S. Shourijeh *, Argyrios Stampas, Shuo-Hsiu Chang, Radha Korupolu and Gerard E. Francisco

Department of Physical Medicine and Rehabilitation, UTHealth Houston McGovern Medical School, Houston, TX 77030, USA

Supplementary Material – S1

Joint Stiffness

Joint stiffness and damping quantify the resistance of spastic muscles to stretch and movement, critical for understanding dynamic impairments. Stiffness reflects resistance to displacement (e.g., muscle lengthening), while damping reflects resistance to velocity (e.g., rapid joint motion). These metrics, derived from muscle-tendon forces and joint kinematics, provide objective measures of spasticity's mechanical impact, surpassing subjective scales like MAS.

$$k_j = -\frac{\partial M_j}{\partial \theta_j} \text{ where } M_j = \sum_{i=1}^{n} r_{ij} F_i^T \tag{S1}$$

Carrying out the partial derivative and employing the chain rule for differentiation leads to

$$k_j = -\sum_{i=1}^{n} \left(\frac{\partial r_{ij}}{\partial \theta_j} F_i^{MT} + r_{ij} k_i^{MT} \frac{\partial \ell_i^{MT}}{\partial \theta_j} \right) \tag{S2}$$

Recalling that $r_{ij} = -\frac{\partial \ell_i^{MT}}{\partial \theta_j}$, Eq. (S2) simplifies further to

$$k_j = -\sum_{i=1}^{n} \left(\frac{\partial r_{ij}}{\partial \theta_j} F_i^{MT} - r_{ij}^2 k_i^{MT} \right) \tag{S3}$$

$$k_i^{MT} = -\frac{\partial F_i^{MT}}{\partial \ell_i^{MT}} \tag{S4}$$

On the other hand, assuming a serial elasticity between tendon and muscle,

$$k_i^{MT} = \left(\frac{1}{k_i^M} + \frac{1}{k_i^T} \right)^{-1} \tag{S5}$$

Where $k_i^M = -\frac{\partial F_i^M}{\partial \ell_i^M}$ and $k_i^T = -\frac{\partial F_i^T}{\partial \ell_i^T}$ (S6)

if the muscle model is assumed to have noncompliant tendon [20,21], tendon stiffness approaches infinity, and MT stiffness will become equal to the muscle stiffness, $k_i^{MT} = k_i^M$, in which case it can be formed analytically [48,49]. Assuming a noncompliant tendon (infinite stiffness) simplifies calculations, though in spasticity, tendon compliance may contribute significantly to joint resistance. In the above equations, k_j is stiffness of joint j, M is joint moment, θ is joint angle, r is the moment arm of muscle-tendon (MT) unit i about joint j, F^{MT} is force of the muscle-tendon unit.

Joint Damping

$$c_j = -\frac{\partial M_j}{\partial \dot{\theta}_j} \text{ where } M_j = \sum_{i=1}^{n} r_{ij} F_i^{MT} \tag{S7}$$

$$c_j = -\sum_{i=1}^{n} \left(\cancel{\frac{\partial r_{ij}}{\partial \dot{\theta}_j}} F_i^{MT} + r_{ij} \frac{\partial F_i^{MT}}{\partial \dot{\theta}_j} \right) \tag{S8}$$

Note that moment arm r_{ij} does not depend on joint velocity $\dot{\theta}_j$, so $\frac{\partial r_{ij}}{\partial \dot{\theta}_j} = 0$; therefore,

$$c_j = -\sum_{i=1}^{n} \left(r_{ij} \frac{\partial F_i^{MT}}{\partial v_i^{MT}} \frac{\partial v_i^{MT}}{\partial \dot{\theta}_j} \right) \tag{S9}$$

Where c_j is joint damping, v is MT velocity, $\dot{\theta}$ is joint angular velocity. The term $c_i^{MT} = -\frac{\partial F_i^{MT}}{\partial v_i^{MT}}$, representing the damping of the muscle-tendon unit, can be calculated analytically, similar to the stiffness case, if the tendon is assumed to be noncompliant.

Quasi-stiffness and damping

$$k_{j,quasi} = -\frac{dM_j}{d\theta_j} \tag{S10}$$

$$c_{j,quasi} = -\frac{dM_j}{d\dot{\theta}_j} \tag{S11}$$
